# Aging well with *Norad*

**DOI:** 10.7554/eLife.45974

**Published:** 2019-03-08

**Authors:** Filipa Carvalhal Marques, Igor Ulitsky

**Affiliations:** 1 Department of Biological RegulationThe Weizmann Institute of ScienceRehovotIsrael

**Keywords:** long noncoding RNA, aging, NORAD, PUMILIO, genomic stability, mitochondria, Human, Mouse

## Abstract

Deleting a long noncoding RNA drives premature aging in mice.

**Related research article** Kopp F, Elguindy MM, Yalvac ME, Zhang H, Chen B, Gillett FA, Lee S, Sivakumar S, Yu H, Xie Y, Mishra P, Sahenk Z, Mendell JT. 2019. PUMILIO hyperactivity drives premature aging of *Norad*-deficient mice. *eLife* **8**:e42650. doi: 10.7554/eLife.42650

Aging is a complex process that affects most living organisms. Over a century of research has revealed that many different intrinsic and extrinsic factors contribute to our bodies growing old, but we are still a long way from a full understanding of all the mechanisms at play. Recent research has implicated molecules that do not code for proteins in this process, such as long noncoding RNAs. For instance, many of these so-called 'lncRNAs' are expressed differently in patients with age-related disorders, such as various forms of neurodegenerative disease and cancer (Rinn and Chang, 2012).

Now, in eLife, Joshua Mendell of the University of Texas Southwestern Medical Center (UTSW) and colleagues – including Florian Kopp as first author – report that losing the long noncoding RNA *Norad* accelerates aging in mice (Kopp et al., 2019). Previous studies have shown that *Norad* is highly conserved in mammals, and that it is abundant in most human cells (Lee et al., 2016; Tichon et al., 2016). The production of *Norad* increases in response to DNA damage and it helps preserve the genetic information as cells divide. *Norad* is mainly found in the cytoplasm, where it can bind two PUMILIO proteins (PUM1 and PUM2) that regulate a variety of targets in the cell, including some involved in cell growth and division (Figure 1). Yet, it was unknown what deleting *Norad* would entail at the level of an organism.

**Figure 1.**
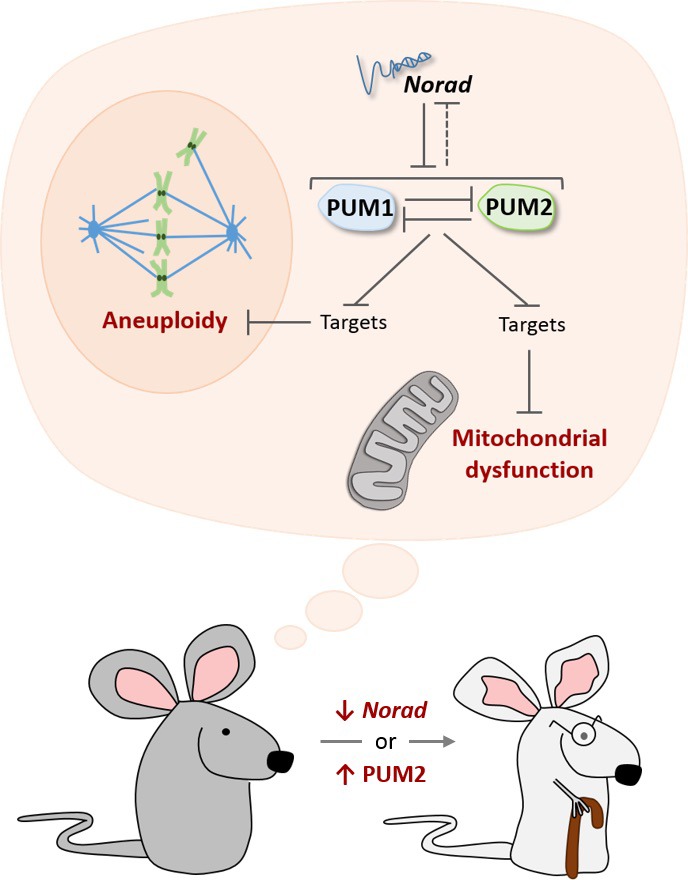
The *Norad*-PUMILIO axis. The long noncoding RNA *Norad* (top) can bind to the PUMILIO proteins PUM1 (pale blue) and PUM2 (pale green) and repress their activity (Lee et al., 2016; Tichon et al., 2016). PUM1 and PUM2 also inhibit each other, and they may repress *Norad* (dotted inhibitory arrow; Goldstrohm et al., 2018). In mice that are genetically engineered to lack *Norad* or to overproduce PUM2, PUMILIO proteins become overactive and strongly inhibit molecules that prevent damage to mitochondria (bottom dark and light gray structure), as well as molecules that prevent cells from acquiring the wrong number of chromosomes (aneuploidy). As a result, these mice show accelerated aging (bottom; Kopp et al., 2019).

To investigate this question, Kopp et al. – who are based at UTSW, the Nationwide Children's Hospital and Ohio State University – engineered mice that lacked *Norad*. As the mutant animals grew, they displayed signs of premature aging: their fur thinned down and grayed faster; their spine showed the abnormal curvature associated with old age; their fat reserves dwindled more quickly; and they died earlier. Mice lacking *Norad* also exhibited several cellular hallmarks of aging (López-Otín et al., 2013). Many of their cells had the wrong number of chromosomes (as previously seen in *Norad*-deficient human cells), as well as mitochondria that were showing defects. This was associated with the repression of PUMILIO targets that keep mitochondria working properly. Strikingly, mice engineered to over-produce PUM2 had similar characteristics, clearly indicating that the hyperactivity of PUMILIO drives the symptoms associated with a lack of *Norad* (Figure 1).

On the flipside, a recent study in humans showed that halving the dose of PUM1 leads to developmental delay and seizures (Gennarino et al., 2018), and reducing the amount of PUM1, PUM2, or both results in smaller body size (Lin et al., 2019). These results suggest that mammals must maintain the activity of PUMILIO proteins within a narrow range in order to remain healthy. In fact, the levels of PUM2 proteins in bulk tissues are indistinguishable between control mice and animals that overexpress *Pum2.* Furthermore, in both *Norad*-deficient and *Pum2*-overexpressing mice, most PUMILIO targets are only affected very mildly, yet the animals exhibit striking phenotypes. Kopp et al. argue that repressing many PUMILIO targets at the same time, even weakly, may trigger the physiological damages observed in these rodents.

Still, it may also be possible that when the *Norad*-PUMILIO axis is disrupted, certain cells or tissues are more likely to stop working properly, which in turn creates a snowball effect for the whole organism. For instance, the expression of *Norad* is highest in the human brain, but it decreases with age in a region that acts as a reservoir to regenerate brain cells (Barry et al., 2015). In simpler organisms, such as the worm *C. elegans*, the nervous system acts on other tissues to coordinate pathways that keep proteins in their normal conformation, but these mechanisms become defective with age (van Oosten-Hawle and Morimoto, 2014). It remains unclear whether the nervous system performs such roles in mammals; yet, these results raise the possibility that interactions between *Norad* and PUMILIO are needed in specific tissues to ward off the effects of age.

*Norad* is PUMILIO’s preferred target, and has several binding sites that are recognized by these proteins. This suggests that this lncRNA can bind a large number of PUMILIO proteins at any given moment, but it is still unclear whether *Norad* tempers the activity of PUM1 and PUM2 simply by competing with other targets of PUMILIO, or if another mechanism is at play (Goldstrohm et al., 2018; Tichon et al., 2018).

Curiously, *Norad* is only found in mammals, whereas the PUMILIO proteins are found in all eukaryotes; it is therefore possible that RNAs with different sequences also buffer the activity of PUMILIO in other species. It remains to be seen how and why mammalian cells rely on *Norad* to tame the activity of these proteins, as opposed to other types of regulatory mechanisms. Ultimately, the work by Kopp et al. sets the ground for further enquiries into the role of noncoding RNAs and RNA-binding proteins in aging, which could potentially yield new therapeutic approaches for diseases brought by old age.
